# Obtaining and Characterizing New Advanced Materials

**DOI:** 10.3390/ma16051881

**Published:** 2023-02-24

**Authors:** Andrei Victor Sandu

**Affiliations:** 1 Department of Technologies and Equipment for Materials Processing, Faculty of Materials Science and Engineering, Gheorghe Asachi Technical University of Iasi, 41 D. Mangeron Blvd., 700050 Iasi, Romania; sav@tuiasi.ro; 2 Romanian Inventors Forum, St. P. Movila 3, 700089 Iasi, Romania; 3 National Institute for Research and Development in Environmental Protection INCDPM, Splaiul Independentei 294, 060031 Bucharest, Romania

**Keywords:** geopolymers, fly ash, thermal insulation, laser sintering, chia seed oil

## Abstract

This editorial highlights the results presented in the second Special Issue dedicated to obtaining and characterizing new materials, wherein one review paper and 13 research articles have been published. The most important field covered is that of materials involved in civil engineering, focusing on geopolymers and insulating materials alongside developing new methods for enhancing the characteristics of different systems. Another important field is that of the materials used for environmental issues, and finally, those involved in human health.

## Introduction

The development of new materials opens up more and more opportunities day by day, and after a successful first Special Issue “Obtaining and Characterizing New Materials”, with the support of editors, we have created a second Special Issue to highlight the latest trends in the broad field of materials engineering.

This second collection, much like the first one, covers a large range of topics. We discuss obtaining and characterizing new materials, from nano- to macro-scale, involving new alloys, ceramics, composites, biomaterials, and polymers, as well as procedures and technologies for enhancing their structures, properties, and functions. In order to choose the future applications of these new materials, we first must understand their structures and know their characteristics by involving modern techniques such as microscopy (SEM, TEM, AFM, STM, etc.), spectroscopy (EDX, XRD, XRF, FTIR, XPS, etc.), mechanical tests (tensile, hardness, elastic modulus, toughness, etc.), and understand their behavior (corrosion, thermal, DSC, STA, DMA, magnetic properties, biocompatibility, in vitro and in vivo).

The most represented of the domains included here is that of construction materials, in which we seek to create sustainable materials with low costs and effective characteristics.

Geopolymers are a type of inorganic polymer that can be made from a wide range of materials and also from industrial wastes. In order to produce cementitious products inside treated soils and improve the mechanical and physical qualities of clayey soils, alkaline activation of industrial waste was posited. This way, the use of geopolymers based on fly ash and ground-granulated blast furnace slag (GGBFS) for soil stabilization increased their strength. S.R. Abdila et al. [1] involved two different types of precursors, performing unconfined compressive strength (UCS) and concluding that GGBFS and fly ash-based geopolymers can successfully be used for soil stabilization [1].

Due to its improved acid resistance compared to regular Portland cement and lower CO2 emissions during the synthesis process, a fly ash geopolymer is suggested as a material for rigid pavement applications. In order to manufacture a fly ash-based geopolymer with the best compressive strength, the authors have sought to improve its formulation. The findings indicate that for fly ash-based geopolymers, the ideal sodium hydroxide concentration, sodium silicate to sodium hydroxide ratio, and solid-to-liquid ratio are, respectively, 10 M, 2.0, and 2.5, with a maximum compressive strength of 47 MPa. With a higher percentage of compressive strength than OPC concrete, the geopolymer is a more durable material for rigid pavement applications because it is based on fly ash, with a percentage of compressive strength loss of 7.38% to 21.94% for OPC concrete [2].

Another study [3] focused on developing a geopolymer based on fly ash; in this study, ladle furnace slag was combined with foam created by pre-foaming polyoxyethylene alkyether sulphate (PAS). At temperatures between 29 and 1000 °C, the performance of a fly ash-slag geopolymer blend with and without PAS foam was examined using a PAS foam-to-paste ratio of 1 and 2. (G-1 and G-2). At 29–1000 °C, the compressive strength of the foamed geopolymer was lower than that of G-0 (36.9–43.1 MPa) (25.1–32.0 MPa for G-1 and 21.5–36.2 MPa for G-2). Heating G-0 decreased its compressive strength by 8.7%, up to 1000 °C compared to unheated samples; however, foamed geopolymer gained compressive strength by 68.5% up to 1000 °C.

In a highly sought after area, that of the road concrete development, L.M. Nicula et al. [4] involved nuclear magnetic resonance (NMR) relaxometry to compare the porosity of three combinations of road concrete that contain blast furnace slag to two mixtures created with conventional ingredients. The samples involved were maintained for 300 freeze–thaw cycles and then compared to control samples. The investigations allowed for the identification of the ideal composition of blast furnace slag to be added to road concrete mixtures. Additionally, using this non-invasive method, it is possible to evaluate the porosity and the development of interior cracks during the freeze–thaw test.

In other aspect of civil engineering, the involvement of wood is crucial. P. Mania et al. [5] managed to densify samples of paulownia clone wood and hornbeam (*Carpinus betulus* L.). The specimens underwent plastic treatment in an ammonia solution before being densified. Following densification, it was possible to measure the wood’s Brinell hardness in each of the three anatomical directions, and its compressive strength in the radial direction. The extent to which wood would swell in water that was liquid and humid (98% RH) was also determined. The densities of hornbeam and paulownia wood increased by 40% and approximately 280%, respectively. For hornbeam and paulownia, the Brinell hardness parallel to the fibers increased by 49 and 390%, and perpendicularly by 80 and 388%. Additionally, it was discovered that the woods’ compressive strength significantly increased in the radial direction. Paulownia wood exhibited 107% swelling, compared to 153% for densified hornbeam wood exposed to water.

For many years, multi-beam box girder bridges have been used extensively worldwide. For this issue, a method for reinforcing embedded steel plate (ESP) was proposed by adding carbon-A/-B glue to the longitudinal joints of old multi-beam box girder bridges [6]. We can conclude that the proposed strengthening method can be used to enhance the mechanical performance of multi-beam box girder bridges and serve as a guide for such bridge reinforcement. Analysis results of the actual bridge and finite element model show that the structural stiffness and load lateral transferring performance between the box girders were improved after ESP strengthening.

Given the current situation, it is necessary to find sustainable and cutting-edge ways of improving the thermal efficiency of projects. An intriguing study [7] shows experimental findings from the testing of a variety of composite thermal insulation materials made from a blend of sheep wool, cellulose, rPET, and rPES fibers. The study’s findings highlight the advantages of utilizing such materials for improving indoor air quality, while also demonstrating their qualities of thermal insulation (the ability to adjust to humidity and reduce concentrations of harmful substances). When compared to conventional thermal insulation materials, the benefits of employing sheep wool composite mattresses in terms of their resistance to insect attack are also shown.

Some researchers [8] discuss the capacity of sheep wool heat-insulating mattresses to simultaneously meet the real need for high-quality air inside living spaces and thermally efficient buildings by cumulatively analyzing efficiency indicators for thermal insulation and indicators of improved air quality. As a result, the results for the coefficient of thermal conductivity and its resistance to heat transfer show that these mattresses are appropriate for use as thermal insulation. The discovered features of permeability to water vapor and the sorption/desorption of water and air show their ability to control the humidity of indoor air, and the resulting decrease in formaldehyde content show their contribution to the improvement of air quality.

On the subject of materials and sustainability, we need to focus on environmental issues and materials’ impact on human health.

In this vein, investigations were conducted on soil samples to perform a quality status assessment, determining pH, texture, structure, and metal concentration, as well as carrying out an assessment of anthropogenic activity by determining the pollution indices of CF (contamination factor). These investigations aimed to determine soil quality, soil environmental risk, and extraction of metals from polluted soils by bioleaching, and aimed to identify influential factors in achieving high remediation yields (potential ecological risk index). Though optimistic, the depollution yield after 12 h of treatment is Cu 29–76%, Pb 10–32%, Cr 39–72%, and Ni 44–68%. The best exposure duration for the bioleaching extraction process can be determined using yield–time correlation equations [9].

Many other parts of the industry are seeking new technologies and materials. To investigate the effects of the powder’s physical characteristics and operating conditions on the bed quality—which is determined by density characteristics, density uniformity, and the flatness of the powder layer—powder spreading in realistic SLS settings was simulated using the discrete element method (DEM) [10]. Based on the response surface methodology, a regression model of the powdering quality was created (RSM). The non-dominated sorting genetic algorithm II (NSGA-II) was utilized to optimize the nylon powder laying quality in the SLS process using an improved multi-objective optimization approach. We offered various optimization plans in accordance with the various process needs. Experiments were used to confirm the validity of multi-objective optimization outcomes for powdering quality.

In another of this publication’s articles [11], the influence of isothermal annealing on the mechanical and microstructural properties of Sn-0.7Cu-1.5Bi solder junctions is discussed. The intermetallic layer thickness at the solder/Cu interface increased by 0.042 m/h for Sn-0.7Cu and 0.037 m/h for Sn-0.7Cu-1.5Bi, according to the results, as the solder/Cu interface cured. With a 1.5 weight percent Bi addition in the reflowed condition and after isothermal annealing, the hardness and shear strength of Sn-0.7Cu dramatically increased.

Innovative materials known as shear thickening fluids (STFs) can be used in smart body armor. The objective of the published paper [12] was to examine how UV light affects STF aging. Artificial aging was used in the experimental inquiry to look into how UV light affects the characteristics of STFs. The highest viscosity of the STFs based on PPG425, PPG2700, and KE-P10 reached 580.7 PaS for the STF425 and 3313 PaS for the STF2700, respectively. Our findings show that STFs are UV light-sensitive and can lose some of their characteristics while being stored.

Building on our knowledge of coatings, J.J.K.Ngouoko et al. [13] studied a glassy carbon electrode (GCE) coated with a sheet of hydroxy-apatite (HA)/L-lysine (Lys) composite material to create an amperometric sensor for the detection of Nile blue A. (NBA). Electrochemical research revealed an increase in the GCE/Lys/HA sensor’s sensitivity to the detection of NBA in solution. Investigations were carried out to elucidate how the pH, scan rate and NBA concentration affected the peak current and potential. The GCE/Lys/HA sensor demonstrated excellent repeatability, selectivity, and an NBA low detection limit of 5.07 108 mol L1 under ideal circumstances. NBA in several water samples was successfully detected using the developed HA/Lys-modified electrode.

In the field of organic chemistry, a novel epoxidized vegetable oil (EVO) from chia seed oil (CSO) has been produced with the intention of use as a plasticizer and compatibilizer in a wide range of eco-friendly goods related to the polymeric industry [14]. Analysis of various parameters revealed that 75 °C and an H_2_O_2_:DB (1.50:1) ratio produced the best epoxidation results. These high values demonstrate that the potential of chia seed oil’s chemical modification to be exploited in the creation of biopolymers is much greater than that of commercially available epoxidized oils such as soybean or linseed oil.

Finally, the last article [15] examined the ways in which hydrochloric acid and brushing affected the surface quality of three flowable composite resins used for direct restoration, concluding that brushing with firm bristles straight after an acidic challenge causes two of the three flowable composite resins to have increased surface roughness. Thirty minutes after acidic aggression, brushing teeth with medium or stiff brushes had no effect on the flowable composite resins’ surface quality.

In conclusion, the published articles under this Special Issue represent an excellent collection of research and review articles, with cutting-edge results that promote sustainable development in the field of materials engineering.
